# Lung Metastasis Mimicking Fingertip Infection

**DOI:** 10.1155/2015/708789

**Published:** 2015-07-07

**Authors:** Salih Soylemez, Murat Demiroglu, Mehmet Ali Yayla, Korhan Ozkan, Bugra Alpan, Harzem Ozger

**Affiliations:** ^1^Department of Orthopaedics and Traumatology, Istanbul Medeniyet University, Goztepe Education and Research Hospital, 34722 Istanbul, Turkey; ^2^Department of Orthopaedics and Traumatology, Istanbul University, Istanbul Faculty of Medicine, 34093 Istanbul, Turkey

## Abstract

Metastasis fingers (acral metastasis) are finding a poor prognosis. Past medical history should be questioned and metastasis from primary tumor should be kept in mind in patients with pain, swelling, and hyperemia in fingers. Successful surgical treatment on acral metastasis does not extend the life expectancy; however, it reduces the patient's pain during his terminal period, saves the functions of the limb, and increases life comfort.

## 1. Introduction

Bone tumors most commonly appear as metastases of primary tumor, like prostate, lung, breast, kidney, and gastrointestinal, different parts of the skeletal system. Malignant tumors are rarely seen in hands. Most common tumors are ganglia and giant cell tumors of the tendon sheets at the hand [1]. The most common primary malignant osseous tumor of the bone is osteosarcoma [2].

Metastasis to bones constituting the hands (acral metastasis) is very rare and associated with poor prognosis. Acral metastasis makes 0.1% of all bone metastases [3].

44% of acral metastases originate from primary lung tumors, while 0.2% of patients with lung cancer present with acral metastasis [4, 5]. Other tumors may present with acral metastasis which can be originated from breast and gastrointestinal system [3, 6, 7]. While extrapulmonary tumors spread within blood, they are first filtered in the lung and liver tissue, so less tumor cells spread in the limbs compared to other parts of the body. Tumor cells originated from lung cancer enter the arterial system where they directly spread to extremities [8].

In this case report, we aimed to present a case of bilateral acral metastasis due to lung cancer and display the importance of different presentation of the primary tumors.

## 2. Case

**Figure 1:**
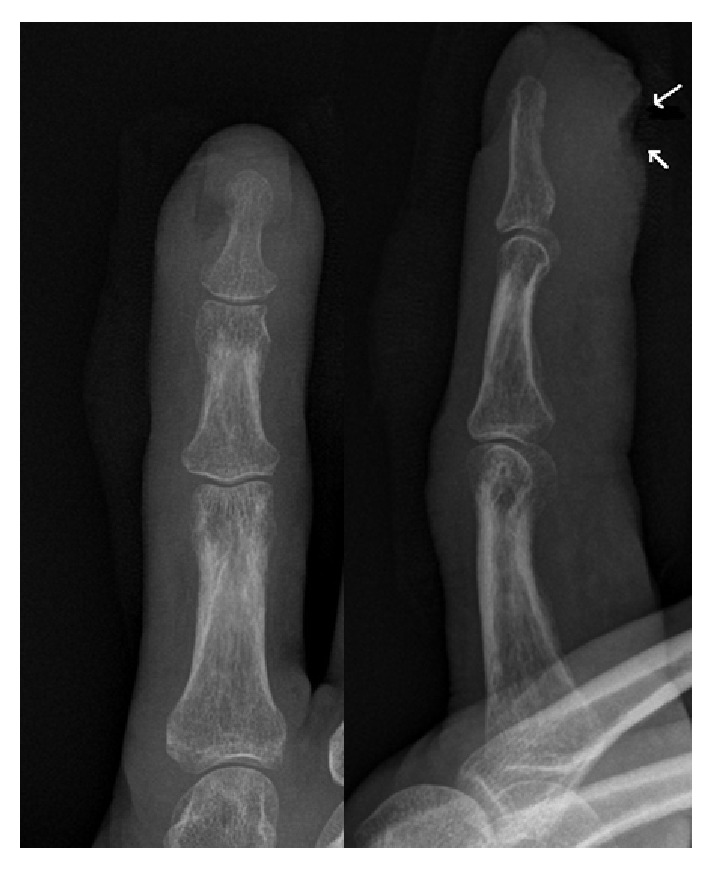
Preoperative anteroposterior and lateral radiographs of the index finger. Arrows: necrotic area in the soft tissues.

A 47-year-old man was admitted to our clinic with complaints of a wound on his right hand's index fingertip (Figure 1). He had intense pain. In his physical examination, the ulcerated lesion 2 × 1 cm wide in the fingertip was revealed. His finger was swollen and hyperemic; putty white discharge was coming out from the wound.

His past medical history revealed that he had been diagnosed with squamous cell lung cancer ten months ago. He had undergone total lobectomy of his left lung. Before consulting our clinic, he was examined at another center and drainage was done with possible diagnosis of finger abscess.

Due to radiolucency detected in his distal phalanx in the radiographies, a true cut biopsy was performed. Squamous cell lung cancer metastasis was determined with biopsy.

Then, the patient underwent amputation to the proximal 1/3 of middle phalanx. On the pathologic examination, proximal surgical margin was found to be free of tumor cells. His sutures were removed on postoperative 10th day and he was pain-free with soft tissue coverage of the finger.

**Figure 2:**
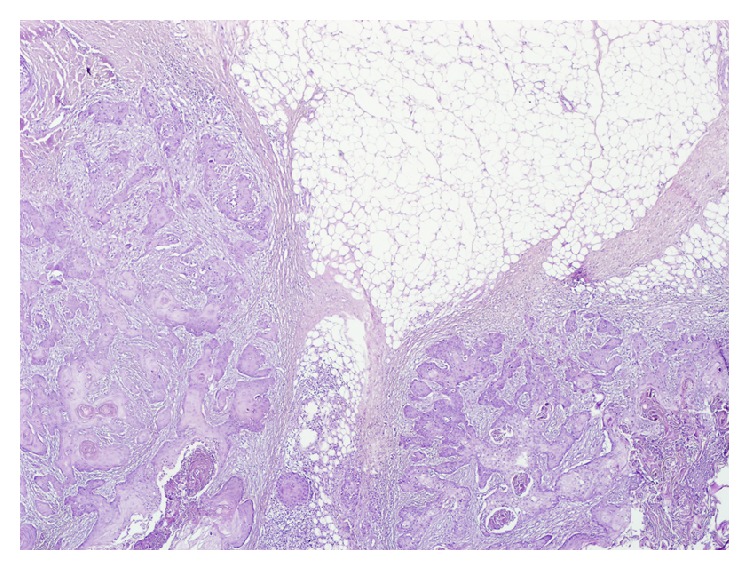
(H&E ×40) Focal keratin pearls and invasive squamous epithelial cluster containing a single cell keratinization.

**Figure 3:**
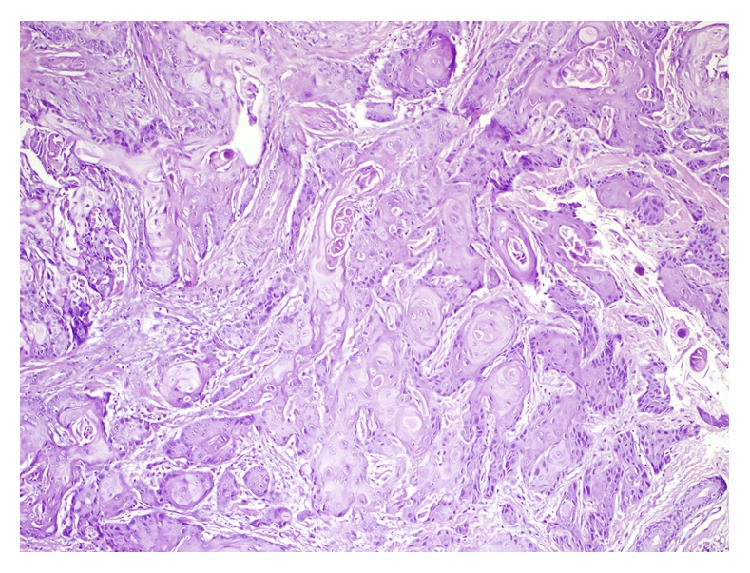
(H&E ×100).

**Figure 4:**
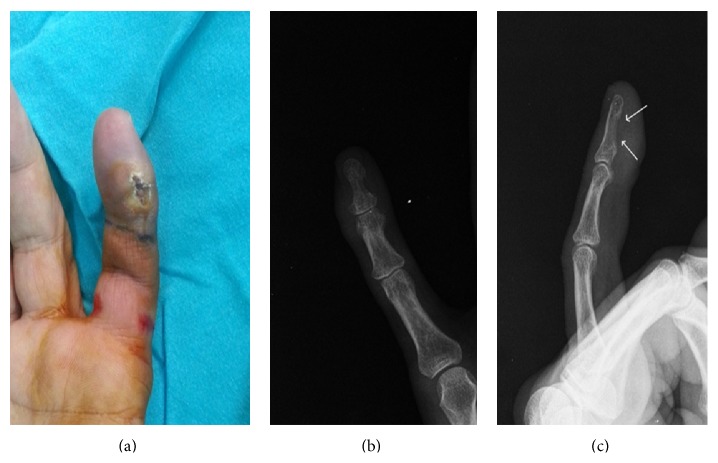
(a) Painful ulcerated lesion on the finger pulp on the left hand's small finger. ((b), (c)) PA and lateral radiographs. (c) Arrows in cortical bone and soft tissue increased radiolucent areas.

Four months after the first amputation, he was admitted again to our clinic for tense pain in his left hand's small finger pulp presenting the same complaints. Examination revealed the radiolucency in the cortex of distal phalanx of the fifth finger (Figure 4). Following a true cut biopsy, diagnosis of a squamous cell carcinoma metastasis was established (Figures 2 and 3).

**Figure 5:**
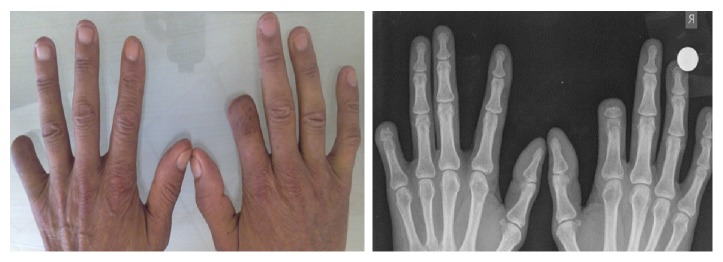
Clinical and radiographic images at postoperative 10th month.

Likewise, the patient underwent amputations to the proximal 1/3 of middle phalanx. After a pathological examination, surgical margin was found clean. Sutures were removed on postoperative 10th day and he was pain-free. In postoperative examinations taken after 10 months after his first visit to clinic, there were no new acral metastases. He was able to use his hands normally during activities (Figure 5).

## 3. Discussion

Acral metastasis is usually associated with advanced lung cancer and very rarely can be the first sign of the carcinoma. It can also mimic infection or an inflammatory disease. Acral metastasis is usually a malignant prognostic sign and median survival time is 6 months [9, 10]. It is usually seen most commonly on the dominant hand and distal phalanx. The most common bony metastasis occurs to the third finger. Bilateral involvement is very rare and has been reported in 9% of cases [5].

Symptoms and signs of acral metastasis are usually similar to benign lesions and patients present with edema, hyperemia, ulcerated lesions, and pain. Physicians should be suspicious for the diagnosis.

Although the majority of patients with these symptoms or signs have benign lesions, like infections and benign tumors (e.g., giant cell tumor of tendon sheath), it is crucial to make biopsy to confirm the diagnosis to prevent unnecessary amputations or inadequate treatment [1, 11].

Past medical history should be questioned and metastasis from primary tumor should be kept in mind in patients with pain, swelling, and hyperemia in fingers.

Bony metastases originated from bronchogenic carcinoma, thyroid, and renal cell carcinoma are usually associated with lytic and destructive lesions of the bone, while prostate cancer is usually blastic in nature. Breast carcinomas can be associated with both lytic and blastic lesions [11].

Treatment of acral metastasis caused by lung cancer is usually palliative due to short life expectancy [12]. The purpose of treatment and surgery in such cases is relieving the symptoms and retrieving function by performing brief period of rehabilitation. Amputation is the most common and recommended method of finger metastasis treatment. On the other hand, in certain cases local radiotherapy and curettage can be preferred as a treatment option [11]. Although, in common literature, life expectancy after acral metastasis is considered as 6 months, it may extend as in our case. Despite that, a successful acral metastasis treatment would not affect life expectancy. It increases life comfort during the terminal period and simplifies patient's daily life activities.

## 4. Conclusion

When assessing finger masses, lung originated metastasis should be considered and the case should be evaluated accordingly. Successful surgical treatment on acral metastasis does not extend the expectancy of life. However, it reduces the patient's pain during his terminal period, saves the functions of the limb, and increases life comfort.
